# Editorial: The model of Ramadan diurnal intermittent fasting: unraveling the health implications, volume II

**DOI:** 10.3389/fnut.2023.1247771

**Published:** 2023-08-23

**Authors:** MoezAlIslam Ezzat Faris, Ismail Laher, Meghit Boumediene Khaled, Hassane Zouhal

**Affiliations:** ^1^Department of Clinical Nutrition and Dietetics, Research Institute of Medical and Health Sciences (RIMHS), University of Sharjah, Sharjah, United Arab Emirates; ^2^Department of Anesthesiology, Pharmacology and Therapeutics, Faculty of Medicine, The University of British Columbia, Vancouver, BC, Canada; ^3^Department of Biology, University of Sidi-Bel-Abbès, Sidi Bel Abbès, Algeria; ^4^Department of Science and Techniques of Physical and Sporting Activities (STAPS), University Rennes, M2S (Laboratoire Mouvement, Sport, Santé), Rennes, France; ^5^Institut International des Sciences du Sport (2I2S), Rennes, France

**Keywords:** intermittent fasting, time-restricted diet, caloric restriction, religious fasting, metabolic health

## Introduction

Intermittent fasting (IF) is rapidly acquiring popularity in both scientific and public communities (1, 2). The practice of IF is considered a non-pharmacological, costless, and safe dietary modification that leads to several health benefits including health promotion and disease prevention, with the latter more pronounced in metabolic and geriatric diseases (3). The diurnal intermittent fasting of Ramadan (RIF) is a period of obligatory fasting observed every year during the holy month of Ramadan by approximately 1.5 billion Muslims and is one of the most widely observed forms of IF (1). Numerous studies conducted over the past seven decades indicate that observing this one-month religious fast, which lasts between 10 and 21 h per day depending on the geographical location and season, confers many metabolic, mental, and physiological advantages (4).

Among the numerous health benefits of RIF are improvements in anthropometric measurements (5, 6), reductions in metabolic syndrome components (7), and enhancements in cardiometabolic risk factors (8). RIF also improves glucose homeostasis (9), reduces inflammatory and proinflammatory cytokines and oxidative stress (10), improves liver function tests (11), and modulates the gene expression of numerous anti-inflammatory, antioxidant, and circadian rhythm genes in healthy individuals (12, 13). RIF downregulates tumor-promoter proteins and upregulates tumor-suppressor proteins in subjects with metabolic syndrome (14, 15). Despite being the form of IF with the most research, there are still significant voids in our understanding of the effects of RIF on healthy individuals. In addition, it is unknown how RIF affects patients with chronic diseases, such as cardiovascular disease, diabetes, and cancer, who choose to observe the fasting month and decline the legal justification to circumvent Ramadan fasting. Despite the extensive research conducted on RIF over the past seven decades (4, 16), more information is required regarding the effects of RIF on various physiological systems and the potential genetic expressions and epigenetic alterations caused by this religious practice. A deeper comprehension of RIF will facilitate the optimization of its practice, the maximization of its health benefits, and the guidance of healthcare providers in advising chronically ill patients on RIF-related matters. This Frontiers Research Topic, “*The model of Ramadan diurnal intermittent fasting: unraveling the health implications-volume 2*”, provides a summary of the current research on the physiological effects of RIF and seeks to provide a comprehensive overview of observational and interventional human studies on RIF.

## Synopsis of the published articles pertaining to this Research Topic

We invited 616 potential contributors to contribute to this Research Topic and received three abstracts and sixteen manuscripts in response. After extensive filtering and evaluation, eight articles were chosen for this Research Topic. These eight articles were written by 213 authors from 26 countries on five continents (Asia, Europe, Africa, North America, and South America), namely, the United Arab Emirates, Saudi Arabia, Qatar, Tunisia, Bahrain, Morocco, Egypt, Jordan, Pakistan, Indonesia, Malaysia, Türkiye, Iran, Bangladesh, Ethiopia, South Africa, Nigeria, Germany, Italy, Canada, Japan, Poland, France, Spain, Mexico, and Brazil. This Research Topic had over 24,000 views and about 4,900 downloads as of July 2023.

## Roky et al.

In Roky et al., Faris ME's research group conducted a systematic review of the published literature regarding the effect of sex differences on disease outcomes for patients who chose to fast despite being exempt from observing Ramadan fasting based on religious law. These patients presented with digestive, renal, diabetic, cardiovascular, epileptic, and neurological disorders. A systematic search of Scopus and PubMed was conducted for clinical and observational studies of both male and female patients that mentioned Ramadan, diabetes, cardiovascular, renal, gastrointestinal, and epilepsy. The authors selected 38 studies involving 25,023 patients, of whom more than half (55.6%) were male, from 381 original articles. In Ramadan fasting patients, 18 studies found sex-based differences in blood glucose, body mass index (BMI), renal colic, mortality, thrombosis, frequency of hypoglycemia, and gastrointestinal diseases. At both the pre-Ramadan baseline and during Ramadan time points, the preponderance of differences between female and male patients was reported. Outside of Ramadan, renal colic, cardiovascular, and gastrointestinal ailments were more prevalent in male patients, while high BMI, thrombosis, and headache were more prevalent in female patients. In the remaining 20 studies, there was no correlation between sex and the effect of Ramadan fasting on the frequency of these diseases or other outcomes. The authors concluded that sex should be given greater consideration as a determining factor for patients observing Ramadan fasting. There appeared to be differences in the frequency and incidence of maladies between male and female patients during Ramadan. A larger emphasis on sex differences in the incidence and progression of diseases during fasting may improve patient care, particularly for those patients willing to fast during Ramadan.

## Sunardi et al.

Sunardi et al. investigated the impact of Ramadan fasting on water consumption patterns and quantity. In addition, they examined how closely Ramadan fasting individuals adhere to daily water intake recommendations. Using a cross-sectional design with a self-administered drinking practices questionnaire and a 7-day fluid record, the water and beverage intake of online-managed participants was determined. The authors examined and analyzed the responses of 380 participants from five universities in Indonesia who filled out the questionnaires. The results indicated that the total amount of water and beverages consumed by the participants during Ramadan fell below the guidelines. Water was the most popular beverage, followed by beverages with added sugar. They discovered a significant difference between water and beverage consumption at *iftar*, at night, and *suhoor*. The most common drinking pattern during Ramadan is 2-2-4-2, but a drinking pattern of 4-2-2 glasses (four glasses at iftar, two glasses at night, and two glasses at suhoor) significantly increased the likelihood of adhering to the recommended fluid intake compared to other patterns. The authors emphasized the significance and necessity of increasing water and fluid intake during Ramadan and adhering to the 4-2-2 drinking pattern to reach the recommended daily water intake.

## Madkour et al.

Madkour et al. takes into account the significant lifestyle and nutritional changes associated with fasting during Ramadan, including physical activity, sleep quality and duration, food groups, and macronutrients in comparison to the pre-and post-fasting days, the Faris research group investigated the metabolomic changes associated with the observance of Ramadan in metabolically healthy subjects with overweight and obesity. The authors hypothesized that Ramadan fasting may be accompanied by metabolomic signatures and biochemical biomarkers that distinguish Ramadan from non-fasting days. The study suggested that Ramadan fasting is associated with a metabolomic signature that reflects the varied daytime nutritional and lifestyle habits and the nighttime dietary modifications. Fatty acids and amino acids metabolites, vitamin metabolites, and caffeine metabolites are the main metabolomic changes observed during the month of Ramadan.

## Khan et al.

In Khan et al., the Faris research group examined alterations in nutritional and lifestyle factors during the RIF month and their associations with sleep quality and duration among fasting individuals from a variety of ethnic and cultural backgrounds. The authors hypothesized that RIF is associated with substantial alterations in the duration, timing, and quality of sleep. They also hypothesized that nutritional and lifestyle changes that occur during the month of fasting are likely to influence changes in sleep quality and duration. Because multiple lifestyle factors are closely interacting with sleep outcomes during Ramadan, the authors used structural equation modeling (SEM) to examine how specific nutritional and lifestyle changes affected three sleep parameters (sleep quality, sleep duration, and sleep disturbances) among approximately 24,500 fasting Muslims during Ramadan in several countries with diverse nutritional and lifestyle habits and cultural backgrounds. Consuming plant-based proteins and engaging in physical activity (PA) was significantly associated with optimal sleep duration (7–9 h) among Muslims who were fasting during the month of Ramadan. Moreover, smoking was associated with both an increase in sleep disturbances and a decrease in sleep quality. Fruits, vegetables, dates, and plant-based proteins appeared to be associated with improved sleep quality. In addition, improved sleep quality was associated with reduced use of electronic devices (i.e., less exposure to blue light) and reduced consumption of delivered foods at night during Ramadan. There were contradictory findings regarding the relationship between the three sleep parameters and dining at home vs. eating out. These findings suggested that increasing the consumption of fruits, vegetables, and plant-based proteins during Ramadan are significant factors that could aid in enhancing the quality of sleep during the month of Ramadan. In addition to engaging in regular physical activity and refraining from smoking, individuals who observe Ramadan fasting may benefit from other essential factors that promote better sleep.

## Alzhrani et al.

Alzhrani et al. explored how, during the month of Ramadan, daily routines are altered in several ways, including the quantity and timing of sleep and physical activity. In the literature, the effects of altered meal timing and frequency are well described, but the effects of variations in the quality and quantity of sleep during Ramadan have received less attention. During Ramadan, there is a shift in the timing of the two primary meals: suhoor is eaten before dawn, and iftar is eaten at dusk. In addition, meals during Ramadan are typically higher in calories than other periods of the year. A study conducted by BaHammam's team in Jeddah, Saudi Arabia, investigated the correlation between changes in dietary intake, sleep patterns, and physical activity during Ramadan in 115 healthy adults [mostly female (83%), in contrast to many previous studies that primarily examined male people]. Anthropometric data, dietary patterns, sleep–wake patterns (related to “morning” or “evening” people and general levels of daytime sleepiness), and levels of physical activity were measured over 3 months at two-time points: 2 months before the beginning of Ramadan and during the last 3 weeks of Ramadan. During Ramadan, there were slight decreases in body weight and BMI, with minimal changes in body fat and visceral fat, and an increase in total caloric intake and carbohydrate consumption. Moreover, there was a shift in the chronotypes of the participants, with an increase in evening chronotypes and a commensurate decrease in morning chronotypes. The scores for daytime sleepiness increased during Ramadan, but total sleep duration and levels of physical activity did not change between the study sites.

## Romdhani et al.

Romdhani et al. conducted a large, multi-national (participants from 44 countries) study of 1,681 athletes investigated the added impact of COVID-19 on the eating habits and sleep patterns of Muslim study participants. The key findings from this retrospective study were that (i) there were decreases in sleep quality and sleep quantity during the COVID-19 lockdown period, (ii) levels of insomnia increased, (iii) sleep quality was decreased in Muslim athletes, (iii) Muslim athletes reported longer daytime naps and more frequent late-night meals, and (iv) there was a decrease in training volumes among Muslim athletes during Ramadan. This study confirmed that the disruption of sleep and exercise patterns that occurs during Ramadan was exacerbated by the COVID-19 lockdown. The findings of the Romdhani et al. study strongly suggest a significant impact of circadian disruptors (e.g., Ramadan, COVID-19 lockdown, and jet lag) on exercise ability, and the authors argue for better sleep management.

## Boujelbane et al.

Boujelbane et al. examined the effects of RIF on cognitive function and sleep parameters in 58 elderly participants (physically sedentary and physically active groups). Before and during the Ramadan fasting month, both groups underwent the same assessment procedure over two sessions using a digital cognitive assessment battery based on validated questionnaires in Arabic: the Pittsburgh sleep quality index, the insomnia severity index, and the Epworth sleepiness scale questionnaires. Executive function, attention, inhibition, associative memory, and recognition memory all improved significantly, particularly in the physically active group, according to this study's findings. Poor sleep quality accompanied by excessive diurnal sleepiness was observed among the two groups. RIF had a negative impact on the sedentary population. The authors of this research highlighted the beneficial effect of physical activity during the Ramadan fasting days on the sleep quality and cognitive performance of the elderly.

## Al-Jafar et al.

To determine the effects of RIF, Al-Jafar et al. explored anthropometric measurements and body composition before and at the end of Ramadan fasting month. Body mass index (BMI), body weight, fat mass (FM), muscle mass (MM), total body water (TBW), waist circumference (WC), hip circumference (HC), and waist-to-hip ratio (WHR) were the primary parameters examined. This article is divided into two major sections: the first section focuses on the LORANS community-based study that was conducted with a broad population in mind. The second section consists of a systematic review and meta-analysis of studies examining the effect of RIF on anthropometric measurements and/or body composition. The meta-analysis included 66 studies, including the LORANS study with a total of 7,496 participants. The targeted population included both healthy and diseased individuals (type 2 diabetes, overweight/obesity, chronic kidney disease, and metabolic syndrome) and the LORANS study participants. Followed by BMI, WHR, WC, MM, FP, HC, FM, and TBW, the most concerning outcome was body weight. In the second/third week of Ramadan, fasting was associated with a reduction in body weight, BMI, WC, and HC. The decline was considerable in the fourth week, peaked immediately after Ramadan, and began to decline 3–6 weeks after the end of Ramadan.

## Outcomes and future directions

The collection of studies demonstrate different health outcomes result from the observance of RIF, with variable effects resulting from the coexistence of RIF practice with different dietary and lifestyle changes among different communities around the world. Even though significant milestones were reached in elucidating the various health-related effects of RIF, there is still much to learn. The use of advanced molecular techniques (including genomics, proteomics, lipidomics, metagenomics, and transcriptomics) will advance our understanding of the various changes that occur during RIF. In addition, examining changes in the human gut microbiome and its capacity to modulate variable biochemical and physiological changes induced by RIF is an area for further exploration. Similarly, the use of artificial intelligence (AI, such as machine learning and neural networks) could provide further insights into the cumulative effects of RIF on human health and disease.

## Conclusion

This Research Topic in Frontiers helps to expand our understanding concerning the impact of RIF on human nutrition, sleep quality, water intake, health, and disease. It opens a path for future research and will stimulate future discussions among academics worldwide.

## Author contributions

All authors listed have made a substantial, direct, and intellectual contribution to the work and approved it for publication.
